# Influence on attitudes and lifestyle due to lockdown amidst COVID-19 pandemic: a perception-based analysis among Bangladeshi residents

**DOI:** 10.1186/s12889-021-12012-z

**Published:** 2021-11-01

**Authors:** Md. Saiful Islam, Md. Asad Ullah, Ummay Soumayia Islam, Sahadat Hossain, Yusha Araf, Anindya Das, Asir Newaz Khan, Nafisa Nawal Islam, Bishajit Sarkar, Abdullah Mohammad Shohael

**Affiliations:** 1 Department of Public Health and Informatics, Jahangirnagar University, Savar, Dhaka, Bangladesh; 2 Centre for Advanced Research Excellence in Public Health, Savar, Dhaka, Bangladesh; 3 Department of Biotechnology and Genetic Engineering, Jahangirnagar University, Savar, Dhaka, Bangladesh; 4 Department of Genetic Engineering and Biotechnology, Shahjalal University of Science and Technology, Sylhet, Bangladesh; 5 Department of Genetic Engineering and Biotechnology, University of Dhaka, Dhaka, Bangladesh; 6 Department of Economics and Social Science, BRAC University, Dhaka, Bangladesh

**Keywords:** COVID-19, Lockdown, Attitude, Lifestyle, Bangladesh

## Abstract

**Background:**

Countrywide lockdown or stay-at-home order has been implemented to slow down the transmission of emergent coronavirus. However, the influence on attitudes and lifestyle due to lockdown amidst the coronavirus disease 2019 (COVID-19) pandemic has been poorly understood. The present study aimed to investigate the influence on attitudes and lifestyle due to lockdown amidst the COVID-19 pandemic among Bangladeshi residents.

**Methods:**

A cross-sectional survey carried out involving 1635 community dwellers across eight divisions in Bangladesh conducted from April 15, 2020 to May 10, 2020. A structured questionnaire incorporating socio-demographic, attitudes towards lockdown and adverse lifestyle amidst lockdown measures was employed to collect data using the Google Forms. Multiple regression analyses were executed to determine the associated factors of positive attitudes towards lockdown and adverse lifestyle.

**Results:**

The mean scores of attitudes towards lockdown were 67.9 (SD = 8.4) out of 85 with an overall correct rate (positive attitudes) of 79.9%; whereas the mean scores of adverse lifestyle amidst lockdown were 16.1 (SD = 4.8) out of 34 with an overall rate of 47.4%. The factors associated with more positive attitudes towards lockdown included being female, divorced, higher educated, and students. Conversely, being male, having no formal education, and rural residence were associated factors of adverse lifestyle amidst the COVID-19 pandemic.

**Conclusions:**

The findings reflect how the COVID-19 lockdown has preciously impacted the attitudes, and lifestyle of Bangladeshi citizens, which will contribute to promoting appropriate measures during a subsequent zonal or complete lockdown.

## Background

The novel coronavirus that causes a severe respiratory disease also known as COVID-19 was first reported by the World Health Organization (WHO) on December 31, 2019 and got recognition as a global pandemic on March 11, 2020 [1, 2]. Since the first outbreak of the disease in Wuhan, China, the infectious virus started its ravaging effect all over the world except Antarctica [3–5]. The major symptoms of COVID-19 include fever, dry cough, fatigue, myalgia, breathing difficulty and dyspnea [6–8]. Lungs are the most affected organs as the virus enters through the angiotensin converting enzyme 2 that are mostly prolific in the type 2 alveolar cells of the lungs [9]. Due to lack of sufficient knowledge about the disease, health care systems from third to first world countries struggled to treat the massive number of infected COVID-19 patients [10]. Since COVID-19 disseminates through social contact and there was no medicine available to cure the disease, billions of people all over the world went under lockdown measures to minimize the transmission rate [11, 12]. The active involvement of every person on earth with respect to testing, isolation, contact tracing, social distancing, home quarantine, self-quarantine, maintaining hygiene and using personal protective equipment has become important to combat COVID-19’s impact as well as give sufficient time for developing treatment strategies [13, 14]. However, due to varying level of knowledge, attitudes and practices towards the COVID-19 pandemic prevention strategies has brought challenges in many countries [10, 15].

The Institute of Epidemiology, Disease Control and Research (IEDCR, the national institute for conducting disease surveillance and outbreak investigation in Bangladesh) confirmed the country’s first cases of coronavirus on March 8, 2020 [16, 17], and since then to safeguard the citizens, the government of Bangladesh declared a countrywide lockdown from March 26, to May 31, 2020 [18–20]. As of November 20, 2020 Bangladesh confirmed 4,41,159 cases with 6305 deaths and 3,56,722 recoveries [21]. Meanwhile, similar partial or complete lockdown measures were created in other countries; they have disrupted the daily routines of almost two-thirds of the world’s population. Since the lockdown enforcement imposes restrictions on people’s rights by refraining them from normal day-to-day activities, its usefulness remains a subject of debate among different groups of people, as evidenced by various poll studies during this pandemic [22]. Thus, it will be useful and timely to understand the specific public perception, activities, and attitudes during the lockdown so governments and policymakers can regulate, recommend, and take necessary steps to avoid any undesired outcome and fulfill their basic needs [23]. In addition, this lockdown has affected people of Bangladesh based on their knowledge, attitude, and practices; those who are older, more educated, employed, have a monthly family income over 30,000 BDT (Bangladeshi Taka), and reside in urban areas showed more positive attitude towards the COVID-19 pandemic [24]. Due to the restrictions of normal routine life activities during the complete countrywide lockdown the public’s perception, attitude, and activities erupted, and therefore, it is crucial to understand their response for implementing further steps to alleviate their grievances in the long run given the uncertainty surrounding the pandemic which is in the process of being under control.

To date, there are very limited studies that have been executed in Bangladesh regarding people’s attitudes or perceptions across socio-demographic conditions during a countrywide lockdown [24, 25]. In this paper, we quantitatively examined how the people of Bangladesh from different socio-economic status (SES) responded including their daily activities, physical and mental conditions during the countrywide lockdown, which in turn impacted the overall effectiveness of this restrictive measure. The present study was the first report on COVID-19, which covers the people living in both urban and rural areas of all divisions in Bangladesh, capturing the holistic dimension of the influence of attitudes and lifestyle due to lockdown of community people. Thus, the present study aimed to investigate the influence on attitudes and lifestyle due to lockdown amidst the COVID-19 pandemic among Bangladeshi residents. The scientific findings of this study will contribute to recommend necessary and emergency steps that are required to avoid any perilous situation during any further zonal or complete countrywide lockdown in the future.

## Methods

### Study design and participants

This was a cross-sectional survey conducted among 1635 community dwellers across eight divisions (i.e., Dhaka, Chittagong, Rajshahi, Khulna, Barishal, Sylhet, Rangpur, and Mymensingh) of Bangladesh covering both urban and rural areas. Data were collected from April 15, to May 10, 2020, when the country was under complete lockdown, which was enforced on March 26, 2020. The study’s target population was Bangladeshi citizens who were house-bound in Bangladesh during the COVID-19 pandemic. The inclusion criteria included being (i) aged 18 years or older; (ii) able to understand Bangla, and (iii) able to complete the entire survey.

#### Sample size calculation

The sample size was calculated using the following Eq. (1):

1 $$ {\displaystyle \begin{array}{l}n=\frac{z^2 pq}{d^2}\\ {}\Rightarrow n=\frac{1.96^2\times 0.5\times \left(1-0.5\right)}{0{.05}^2}\\ {}\Rightarrow n=384.16\approx 384\end{array}} $$

Here,

*n* = number of samples

*z* = 1.96 (95% confidence level)

*p* = prevalence estimate (50% or 0.5) (as no study found)

*q* = (1-*p*)

*d* = precision limit or proportion of sampling error (0.05)

As there is no prior similar study focusing on the influence on attitudes and lifestyle due to lockdown amidst the COVID-19 pandemic in Bangladesh, we made the best assumption (p) for the present study would be 50%. Assuming a 10% non-response rate, a sample size of 423.5 ≈ 424 participants was estimated. Our sample size exceeded this estimate.

#### Data collection procedures

A self-reported questionnaire incorporating in the Google Forms was employed to survey participants. A total of 24 research assistants who completed their graduation in health and life sciences along with clinicians and statisticians approached the participants to take part in the survey. The age-stratified (over 18 years) convenient sampling method utilized to recruit the participants. In sum, 1635 participants took part in the survey prior to informed consents.

#### Measures

A structured questionnaire incorporating three sections (i.e., socio-demographic information, attitudes towards lockdown, and adverse lifestyle amidst lockdown measures – see below) was utilized to collect data during the data collection periods. At first, a draft questionnaire was prepared based on extensive literature review [26–31].Then it was piloted among 20 individuals in an urban slum of Dhaka city, and then the questionnaire was finalized after checking of its validity by the academic experts knowledgeable in this area. These pilot data were excluded from the final analysis.

### Socio-demographic information

Some basic information of participants was recorded during the survey including age (later categorized: 18–25 years and over 25 years), gender (male/female), Religion (Muslim/Hindu/Buddhist/Christian), marital status (unmarried/married/divorced), and residence (rural/urban). In addition, participants’ education, occupation and resident division were also asked.

### Attitudes towards lockdown measures

The attitudes towards lockdown were measured using structured questions based on the WHO and the Centers for Disease Control and Prevention (CDC) guidelines [26–31]. This section consisted of 17-item questions regarding the positive attitudes towards the lockdown (e.g., “*Lockdown can prevent community spreading,*” see details in Table 2) with a five-point Likert scale ranging from 1 (“*Strongly disagree*”) to 5 (“*Strongly agree*”), and yielding total scores ranging from 17 to 85. An overall greater score indicates more positive attitudes towards the lockdown. The Cronbach’s alpha coefficient of the attitudes towards the lockdown measures was 0.73 which indicates acceptable internal consistency of the reliability (as acceptable value > 0.6) [32, 33].

### Adverse lifestyle amidst lockdown measures

The adverse lifestyle measures were adopted from previous literature based on potential problems creating on daily life while lockdown or in home quarantine [29–31]. This section included 17-item questions (7-item for positive and 10-item for negative, see details in Table 5) concerning the problems due to lockdown with three possible responses including: “*Yes,*” “*No,*” and “*Maybe*”. For positive questions, the responses were coded as 0 = *Yes*, 1 = *Maybe*, and 2 = *No* (e.g., “*I have enough storage of food in my home*”). Conversely, reverse coding as 2 = *Yes*, 1 = *Maybe*, and 0 = *No* incorporated for negative questions (e.g., “*I am smoking more than ever before”*). The total score ranges from 0 to 34, with an overall higher score indicates a greater adverse lifestyle amidst lockdown. The Cronbach’s alpha coefficient of the adverse lifestyle measures was 0.64 which indicates acceptable internal consistency of the reliability (as acceptable value > 0.6) [32, 33].

#### Data analyses

All statistical analyses were performed using the two analytical software (i.e., IBM SPSS Statistics version 25.0, and STATA version 13.0). Descriptive statistics (e.g., frequencies, percentages, means, standard deviations, etc.) and some first-order analyses (e.g., Chi-square tests, Fisher’s exact tests, etc.) were computed. The reliability of the measures of attitudes towards lockdown and adverse lifestyle were evaluated using Cronbach alpha [32, 33]. In addition, inferential statistics including t-tests or one-way ANOVA tests were performed to determine significant relations of the mean attitudes towards lockdown and adverse lifestyle amidst lockdown scores with all examined variables. Finally, variables that significantly differed in terms of attitudes towards lockdown and adverse lifestyle amidst lockdown scores, were included in multiple regression analysis with attitudes towards lockdown and adverse lifestyle amidst lockdown measures, respectively as the independent variables. A *p*-value less than .05 was considered as significant for all statistical tests.

## Results

### General characteristics of participants

The samples comprised 1635 participants with their mean age 29.0 years (SD = 12.2) and their ages ranged from 18 to 82 years. The majority of the participants were males (61.5%), were unmarried (61.3%), had higher secondary education (10 to 12 grades) (63.8%), were students (57.2%), resided in urban areas (59.0%), and were Muslim (84.5%) (Table 1). Besides, a sizeable majority resided in Dhaka division (32.5%).

**Table 1:** General characteristics of participants (*N* = 1635)

Characteristics	*n*	(%)
Gender		
Male	1005	(61.5)
Female	630	(38.5)
Age		
Young (18–25 years)	1015	(62.1)
Older (> 25 years)	620	(37.9)
Religion		
Muslim	1381	(84.5)
Hindu	240	(14.7)
Buddhist	14	(0.9)
Marital status		
Unmarried	1002	(61.3)
Married	619	(37.9)
Divorced	14	(0.9)
Education		
No formal education	59	(3.6)
Primary	67	(4.1)
Secondary	180	(11.0)
Higher Secondary	1043	(63.8)
University	253	(15.5)
Higher education	33	(2.0)
Occupation		
Student	936	(57.2)
Housewife	191	(11.7)
Service Holder (Govt.)	226	(13.8)
Business	145	(8.9)
Unemployed/other	137	(8.4)
Residence		
Rural	670	(41.0)
Urban	965	(59.0)
Resident division		
Dhaka	532	(32.5)
Chittagong	384	(23.5)
Rajshahi	86	(5.3)
Khulna	322	(19.7)
Barishal	89	(5.4)
Sylhet	37	(2.3)
Rangpur	32	(2.0)
Mymensingh	153	(9.4)

### Attitudes towards lockdown

The mean scores of attitudes towards lockdown were 67.9 (SD = 8.4) out of 85 with an overall correct rate (positive attitudes) of 79.9%. Table 2 summarized the distribution of each item attitudes-related questions along with gender differences. The participants’ attitudes score was significantly higher among (i) females vs. males (M = 68.9, SD = 7.0 vs. M = 67.3, SD = 9.1, *p* < .001), (ii) divorced vs. unmarried (M = 72.7, SD = 5.0 vs. M = 67.6, SD = 8.8, *p* = 0.023), (iii) participants having higher vs. secondary levels of education (M = 71.8, SD = 5.2 vs. M = 66.4, SD = 8.2, *p* = .023), (iv) housewives vs. those in business (M = 69.5, SD = 6.5 vs. M = 64.8, SD = 9.2, *p* < .001), (v) participants living in urban vs. rural area (M = 68.2, SD = 8.4 vs. M = 67.4, SD = 8.4, *p* = .04) (Table 3).

**Table 2:** Attitudes towards lockdown and gender difference of participants

Characteristics	Total *N* = 1635		Male		Female		χ^2^	*df*	*P*-value
	*n*	(%)	*n*	(%)	*n*	(%)			
The government should provide food and necessary equipment to the unprivileged people									
Strongly agree	1196	(73.1)	723	(71.9)	473	(75.1)	15.35	4	.004
Agree	207	(12.7)	115	(11.4)	92	(14.6)			
Neutral	109	(6.7)	82	(8.2)	27	(4.3)			
Disagree	44	(2.7)	30	(3.0)	14	(2.2)			
Strongly disagree	79	(4.8)	55	(5.5)	24	(3.8)			
Lockdown can prevent community spreading									
Strongly agree	393	(24.0)	248	(24.7)	145	(23.0)	11.66	4	.020
Agree	314	(19.2)	193	(19.2)	121	(19.2)			
Neutral	230	(14.1)	160	(15.9)	70	(11.1)			
Disagree	329	(20.1)	197	(19.6)	132	(21.0)			
Strongly disagree	369	(22.6)	207	(20.6)	162	(25.7)			
Lockdown can increase crime in the city									
Strongly agree	284	(17.4)	169	(16.8)	115	(18.3)	13.77	4	.008
Agree	361	(22.1)	224	(22.3)	137	(21.7)			
Neutral	293	(17.9)	196	(19.5)	97	(15.4)			
Disagree	238	(14.6)	160	(15.9)	78	(12.4)			
Strongly disagree	459	(28.1)	256	(25.5)	203	(32.2)			
Lockdown will make the environment less polluted than ever									
Strongly agree	1111	(68.0)	695	(69.2)	416	(66.0)	15.66	4	.004
Agree	326	(19.9)	176	(17.5)	150	(23.8)			
Neutral	108	(6.6)	80	(8.0)	28	(4.4)			
Disagree	36	(2.2)	21	(2.1)	15	(2.4)			
Strongly disagree	54	(3.3)	33	(3.3)	21	(3.3)			
Lockdown can be the reason for various animal death for want of food									
Strongly agree	677	(41.4)	391	(38.9)	286	(45.4)	13.19	4	.010
Agree	488	(29.8)	294	(29.3)	194	(30.8)			
Neutral	251	(15.4)	172	(17.1)	79	(12.5)			
Disagree	137	(8.4)	91	(9.1)	46	(7.3)			
Strongly disagree	82	(5.0)	57	(5.7)	25	(4.0)			
Lockdown will create all sorts of medical therapy problems									
Strongly agree	436	(26.7)	265	(26.4)	171	(27.1)	10.81	4	.029
Agree	645	(39.4)	375	(37.3)	270	(42.9)			
Neutral	358	(21.9)	238	(23.7)	120	(19.0)			
Disagree	126	(7.7)	76	(7.6)	50	(7.9)			
Strongly disagree	70	(4.3)	51	(5.1)	19	(3.0)			
Lockdown will create workout problems									
Strongly agree	828	(50.6)	498	(49.6)	330	(52.4)	5.42	4	.247
Agree	485	(29.7)	291	(29.0)	194	(30.8)			
Neutral	188	(11.5)	127	(12.6)	61	(9.7)			
Disagree	82	(5.0)	55	(5.5)	27	(4.3)			
Strongly disagree	52	(3.2)	34	(3.4)	18	(2.9)			
Lockdown will create problems in transferring money									
Strongly agree	466	(28.5)	277	(27.6)	189	(30.0)	18.12	4	.001
Agree	562	(34.4)	338	(33.6)	224	(35.6)			
Neutral	353	(21.6)	248	(24.7)	105	(16.7)			
Disagree	153	(9.4)	91	(9.1)	62	(9.8)			
Strongly disagree	101	(6.2)	51	(5.1)	50	(7.9)			
People are using entertainment sources a lot									
Strongly agree	766	(46.9)	432	(43.0)	334	(53.0)	33.99	4	< .001
Agree	449	(27.5)	266	(26.5)	183	(29.0)			
Neutral	224	(13.7)	160	(15.9)	64	(10.2)			
Disagree	106	(6.5)	81	(8.1)	25	(4.0)			
Strongly disagree	90	(5.5)	66	(6.6)	24	(3.8)			
Couples who are going to be married soon are feeling insecure about their marriage									
Strongly agree	706	(43.2)	416	(41.4)	290	(46.0)	8.76	4	.067
Agree	418	(25.6)	248	(24.7)	170	(27.0)			
Neutral	292	(17.9)	194	(19.3)	98	(15.6)			
Disagree	105	(6.4)	70	(7.0)	35	(5.6)			
Strongly disagree	114	(7.0)	77	(7.7)	37	(5.9)			
Lockdown will create an economic crisis for the country									
Strongly agree	1034	(63.2)	643	(64.0)	391	(62.1)	5.18	4	.269
Agree	407	(24.9)	235	(23.4)	172	(27.3)			
Neutral	130	(8.0)	88	(8.8)	42	(6.7)			
Disagree	20	(1.2)	11	(1.1)	9	(1.4)			
Strongly disagree	44	(2.7)	28	(2.8)	16	(2.5)			
Pregnant women will suffer a lot for getting troubled in regular checkups									
Strongly agree	1009	(61.7)	607	(60.4)	402	(63.8)	7.81	4	.099
Agree	418	(25.6)	252	(25.1)	166	(26.3)			
Neutral	125	(7.6)	88	(8.8)	37	(5.9)			
Disagree	36	(2.2)	26	(2.6)	10	(1.6)			
Strongly disagree	47	(2.9)	32	(3.2)	15	(2.4)			
Health workers will be at high risk to work in the time of lockdown									
Strongly agree	1038	(63.5)	625	(62.2)	413	(65.6)	8.77	4	.067
Agree	386	(23.6)	237	(23.6)	149	(23.7)			
Neutral	133	(8.1)	96	(9.6)	37	(5.9)			
Disagree	41	(2.5)	22	(2.2)	19	(3.0)			
Strongly disagree	37	(2.3)	25	(2.5)	12	(1.9)			
Blood donation can be a serious issue because the donor can be a carrier									
Strongly agree	927	(56.7)	546	(54.3)	381	(60.5)	18.78	4	.001
Agree	340	(20.8)	212	(21.1)	128	(20.3)			
Neutral	171	(10.5)	129	(12.8)	42	(6.7)			
Disagree	134	(8.2)	76	(7.6)	58	(9.2)			
Strongly disagree	63	(3.9)	42	(4.2)	21	(3.3)			
The punishment for breaking lockdown should be very strict like in European and American countries									
Strongly agree	993	(60.7)	596	(59.3)	397	(63.0)	20.23	4	< .001
Agree	384	(23.5)	219	(21.8)	165	(26.2)			
Neutral	167	(10.2)	123	(12.2)	44	(7.0)			
Disagree	52	(3.2)	38	(3.8)	14	(2.2)			
Strongly disagree	39	(2.4)	29	(2.9)	10	(1.6)			
The government. Should increase the time of the lockdown for the safety of its people									
Strongly agree	889	(54.4)	543	(54.0)	346	(54.9)	9.25	4	.055
Agree	389	(23.8)	223	(22.2)	166	(26.3)			
Neutral	211	(12.9)	145	(14.4)	66	(10.5)			
Disagree	81	(5.0)	49	(4.9)	32	(5.1)			
Strongly disagree	65	(4.0)	45	(4.5)	20	(3.2)			
Online classes can be the solution to decrease the educational hindrance during lockdown									
Strongly agree	495	(30.3)	284	(28.3)	211	(33.5)	18.61	4	.001
Agree	403	(24.6)	234	(23.3)	169	(26.8)			
Neutral	341	(20.9)	210	(20.9)	131	(20.8)			
Disagree	163	(10.0)	109	(10.8)	54	(8.6)			
Strongly disagree	233	(14.3)	168	(16.7)	65	(10.3)

**Table 3:** Association between socio-demographic characteristics and mean scores of adverse lifestyle and attitudes

Characteristics	Attitudes					Adverse lifestyle				
	*Mean*	*(SD)*	*95% CI*	*t/F*	*p*-value	*Mean*	*(SD)*	*95% CI*	*t/F*	*p*-value
Gender										
Male	67.3	(9.1)	[66.7, 67.8]	14.81	< .001	16.6	(4.7)	[16.3, 16.9]	35.50	< .001
Female	68.9	(7.0)	[68.3, 69.4]			15.2	(4.8)	[14.8, 15.5]		
Age										
Young (18–25 years)	67.8	(8.7)	[67.3, 68.4]	0.04	.835	16.4	(4.8)	[16.1, 16.7]	12.69	< .001
Older (> 25 years)	67.9	(7.9)	[67.3, 68.6]			15.5	(4.7)	[15.1, 15.9]		
Marital status										
Unmarried	67.6	(8.8)	[67.0, 68.1]	3.78	.023	16.3	(4.9)	[16.0, 16.6]	5.03	.007
Married	68.3	(7.7)	[67.7, 68.9]			15.6	(4.7)	[15.3, 16.0]		
Divorced	72.7	(5.0)	[69.8, 75.6]			14.5	(2.9)	[12.8, 16.2]		
Education										
No formal education	70.9	(5.7)	[69.4, 72.4]	4.53	< .001	18.0	(4.0)	[16.9, 19.0]	6.80	< .001
Primary	66.5	(8.6)	[64.4, 68.5]			17.0	(4.3)	[15.9, 18.0]		
Secondary	66.4	(8.2)	[65.2, 67.6]			16.3	(4.5)	[15.7, 17.0]		
Higher secondary	67.8	(8.5)	[67.3, 68.4]			16.2	(4.8)	[15.9, 16.5]		
University	68.2	(8.6)	[67.2, 69.3]			14.8	(4.8)	[14.2, 15.4]		
Higher education	71.8	(5.2)	[69.9, 73.6]			14.9	(4.4)	[13.4, 16.5]		
Occupation										
Student	67.9	(8.4)	[67.3, 68.4]	7.29	< .001	16.4	(4.8)	[16.1, 16.7]	3.99	< .001
Housewife	69.5	(6.5)	[68.5, 70.4]			15.2	(5.1)	[14.5, 16.0]		
Service holder	68.6	(8.2)	[67.5, 69.7]			15.5	(4.8)	[14.8, 16.1]		
Business	64.8	(9.2)	[63.3, 66.3]			15.5	(4.2)	[14.8, 16.2]		
Unemployed/ other	67.9	(8.9)	[66.4, 69.4]			16.5	(4.4)	[15.8, 17.3]		
Residence										
Rural	67.4	(8.4)	[66.7, 68.0]	4.24	.040	16.8	(4.7)	[16.5, 17.2]	31.98	< .001
Urban	68.2	(8.4)	[67.7, 68.8]			15.5	(4.8)	[15.2, 15.8]		

*SD* Standard deviation, *CI* Confidence interval

Finally, factors that were statistically significant in the group difference analyses (t-tests/ANOVA) were included in a multiple regression analysis (see Table 4). The more positive attitudes were positively associated with the female gender, being divorced, having higher education, and students. Consequently, the residence was not significant in the multiple regression analysis. The regression model predicted 3.4% of the variance in attitudes towards lockdown [*F*_(13, 1621)_ = 5.47, *p* < .001].

**Table 4:** Multiple regression analysis predicting attitudes toward lockdown and adverse lifestyle

Characteristics	Attitudes^a^			Adverse lifestyle^b^		
	*B*	*SE*	*β*	*B*	*SE*	*β*
Gender						
Male	−1.06	0.47	−0.06*	1.52	0.27	0.15***
Female			^c^			^c^
Age						
Young (18–25 years)				0.70	0.43	0.07
Older (> 25 years)						^c^
Marital status						
Unmarried	−5.83	2.30	−0.34*	1.70	1.30	0.17
Married	−4.11	2.27	−0.24	1.44	1.30	0.15
Divorced			^c^			^c^
Education						
No formal education	−0.59	1.86	−0.01	2.08	1.05	0.08*
Primary	−4.57	1.80	−0.11*	1.11	1.02	0.05
Secondary	−5.10	1.60	−0.19**	0.82	0.91	0.05
Higher secondary	−3.85	1.55	−0.22*	−0.03	0.89	−0.00
University	−3.37	1.54	−0.15*	−0.55	0.87	− 0.04
Higher education			^c^			^c^
Occupation						
Housewife	−0.49	1.08	− 0.02	− 0.16	0.63	− 0.01
Service holder	− 0.70	0.98	− 0.03	− 0.11	0.59	− 0.01
Business	−4.12	1.06	−0.14***	− 0.97	0.63	− 0.06
Unemployed/ other	−1.84	1.02	−0.06	0.22	0.60	0.01
Student			^c^			^c^
Residence						
Rural	−0.63	0.45	−0.04	0.98	0.26	0.10***
Urban			^c^			^c^

*B* Unstandardized regression coefficient, *SE* Standard error, *β* Standardized regression coefficient

*p < .05,**p < .01, ***p < .001

^a^Model summery (Attitudes): Covariates: Gender, Marital status, Education, Occupation and Residence; *F*_(13, 1621)_ = 5.47, *p* < .001, *R*^2^_Adj_ = .034

^b^Model summery (Adverse lifestyle): Covariates: Gender, Age, Marital status, Education, Occupation and Residence; *F*_(14, 1620)_ = 7.76, *p* < .001, *R*^2^_Adj_ = .055

^c^Reference category

### Adverse lifestyle amidst lockdown

The mean scores of adverse lifestyle amidst lockdown were 16.1 (SD = 4.8) out of 34 with an overall rate of 47.4%. The distribution of each item’s adverse lifestyle-related question along with gender differences is presented in Table 5. The participants’ adverse lifestyle score was significantly higher among (i) males vs. females (M = 16.6, SD = 4.7 vs. M = 15.2, SD = 4.8, *p* < .001), (ii) young vs. older (M = 16.4, SD = 4.8 vs. M = 15.5, SD = 4.7, *p* < .001), (iii) unmarried vs. divorced (M = 16.3, SD = 4.9 vs. M = 14.5, SD = 2.9, *p* = .007), (iv) participants having no formal education vs. higher education (M = 18.0, SD = 4.0 vs. M = 14.9, SD = 4.4, *p* < .001), (v) unemployed vs. housewife (M = 16.5, SD = 4.4 vs. M = 15.2, SD = 5.1, *p* < 0.001), (vi) participants living in rural vs. urban area (M = 16.8, SD = 4.7 vs. M = 15.5, SD = 4.8, *p* < .001) (Table 3).

**Table 5:** Adverse lifestyle and gender difference of participants

Characteristics	Total *N* = 1635		Male		Female		χ^2^	*df*	*P*-value
	*n*	(%)	*n*	(%)	*n*	(%)			
I have enough storage of food in my home									
Yes	975	(59.6)	548	(54.5)	427	(67.8)	28.51	2	< .001
No	439	(26.9)	307	(30.5)	132	(21.0)			
Maybe	221	(13.5)	150	(14.9)	71	(11.3)			
I have sufficient medicine at home									
Yes	851	(52.0)	483	(48.1)	368	(58.4)	16.87	2	<.001
No	593	(36.3)	392	(39.0)	201	(31.9)			
Maybe	191	(11.7)	130	(12.9)	61	(9.7)			
I have maintained the lockdown properly									
Yes	1255	(76.8)	741	(73.7)	514	(81.6)	13.47	2	.001
No	189	(11.6)	130	(12.9)	59	(9.4)			
Maybe	191	(11.7)	134	(13.3)	57	(9.0)			
I am saving resources for the future									
Yes	710	(43.4)	421	(41.9)	289	(45.9)	2.76	2	.252
No	735	(45.0)	461	(45.9)	274	(43.5)			
Maybe	190	(11.6)	123	(12.2)	67	(10.6)			
I feel mental peace because of not having a workload									
Yes	611	(37.4)	369	(36.7)	242	(38.4)	3.08	2	.214
No	888	(54.3)	543	(54.0)	345	(54.8)			
Maybe	136	(8.3)	93	(9.3)	43	(6.8)			
My relationship with my closest one has improved more than ever									
Yes	695	(42.5)	389	(38.7)	306	(48.6)	18.90	2	< .001
No	627	(38.3)	424	(42.2)	203	(32.2)			
Maybe	313	(19.1)	192	(19.1)	121	(19.2)			
I use a mask when my family members gather together									
Yes	652	(39.9)	378	(37.6)	274	(43.5)	13.81^a^	2	.001
No	956	(58.5)	603	(60.0)	353	(56.0)			
Maybe	27	(1.7)	24	(2.4)	3	(.5)			
I very much feel bored									
Yes	1055	(64.5)	681	(67.8)	374	(59.4)	14.12	2	.001
No	415	(25.4)	224	(22.3)	191	(30.3)			
Maybe	165	(10.1)	100	(10.0)	65	(10.3)			
I am mentally sick									
Yes	394	(24.1)	262	(26.1)	132	(21.0)	5.65	2	.059
No	1123	(68.7)	674	(67.1)	449	(71.3)			
Maybe	118	(7.2)	69	(6.9)	49	(7.8)			
I am using social media more than anything									
Yes	989	(60.5)	633	(63.0)	356	(56.5)	10.41	2	.005
No	533	(32.6)	298	(29.7)	235	(37.3)			
Maybe	113	(6.9)	74	(7.4)	39	(6.2)			
I am smoking more than ever before									
Yes	141	(8.6)	105	(10.4)	36	(5.7)	11.15	2	.004
No	1456	(89.1)	876	(87.2)	580	(92.1)			
Maybe	38	(2.3)	24	(2.4)	14	(2.2)			
I have found sudden changes in my sleeping pattern									
Yes	921	(56.3)	570	(56.7)	351	(55.7)	5.24	2	.073
No	596	(36.5)	374	(37.2)	222	(35.2)			
Maybe	118	(7.2)	61	(6.1)	57	(9.0)			
My family members have broken the lockdown order									
Yes	388	(23.7)	243	(24.2)	145	(23.0)	0.32	2	.854
No	1141	(69.8)	698	(69.5)	443	(70.3)			
Maybe	106	(6.5)	64	(6.4)	42	(6.7)			
My productivity has decreased									
Yes	747	(45.7)	473	(47.1)	274	(43.5)	2.12	2	.346
No	688	(42.1)	410	(40.8)	278	(44.1)			
Maybe	200	(12.2)	122	(12.1)	78	(12.4)			
I feel insecure about my safety									
Yes	962	(58.8)	581	(57.8)	381	(60.5)	1.75	2	.417
No	580	(35.5)	362	(36.0)	218	(34.6)			
Maybe	93	(5.7)	62	(6.2)	31	(4.9)			
I easily get distracted (working slower than normal)									
Yes	782	(47.8)	472	(47.0)	310	(49.2)	2.87	2	.238
No	693	(42.4)	425	(42.3)	268	(42.5)			
Maybe	160	(9.8)	108	(10.7)	52	(8.3)			
My communication over the phone has increased									
Yes	1083	(66.2)	672	(66.9)	411	(65.2)	0.78	2	.677
No	468	(28.6)	280	(27.9)	188	(29.8)			
Maybe	84	(5.1)	53	(5.3)	31	(4.9)			

^a^Fisher’s Exact Test

Finally, factors that were statistically significant in the group difference analyses (t-tests/ANOVA) were included in a multiple regression analysis (see Table 4). The adverse lifestyle was positively associated with male gender, having no formal education, and rural residence. Consequently, age, marital status, and occupation were not significant in the multiple regression analysis. The regression model predicted 5.5% of the variance in adverse lifestyle [*F*_(14, 1620)_ = 7.76, *p* < .001].

## Discussion

In the following study, we aimed to find out the influence on attitude and lifestyle practice due to lockdown amidst the COVID-19 pandemic among Bangladeshi residents. Lifestyles are interlinked with beliefs and attitudes, hence all of these factors play an important role in promoting health both physical and mental [34]. Since Bangladesh stands in the 18th position in terms of confirmed cases of COVID-19 across the globe as of October 24, 2020 [35], it is crucial to ensure and implement quality health promotion strategy among people to mitigate the rising physical and psychological complications during and after COVID-19.

According to our study, a desired number of participants (79.9%) showed positive attitudes toward overcoming COVID-19 during the time of lockdown which is greater than other studies conducted in Bangladesh (62.3%) and Pakistan (59.2%) [5, 36]. However, an epidemiological survey accomplished in North-Central Nigeria stated that, nearly 80% participants positively supported government measures to control the pandemic in their region [1]. There were several variables among participants that showed a more positive attitude toward the pandemic in our findings. In terms of gender differences, our study showed that positive attitude was significantly higher in females (M = 68.9, SD = 7.0) compared to males (M = 67.3, SD = 9.1) which shows similarity with several studies conducted in Saudi Arabia population (female: M = 28.35, SD = 2.32 vs. male M = 28.06, SD = 3.27) [37]. Again, in terms of educational status, participants who have a higher education level (M = 71.8, SD = 5.2) showed more positivity than participants having secondary level education (M = 66.4, SD = 8.2) towards attitude. The findings show similarity with a study previously conducted on Bangladeshi people during COVID-19 outbreak, which was for higher education (74.1%) vs. secondary level education (52.2%) [24]. Another previous study of Pakistan about knowledge, attitude, practice and risk factors regarding COVID-19, also reported slightly higher scores for post-graduation students than undergraduate students [38].

The present study also showed that housewives (M = 69.5, SD = 6.5) have a higher positive attitude compared to the business occupation (M = 64.8, SD = 9.2) and is consistent with a previously conducted study in Bangladesh [24]. In contrast, studies in Pakistan and China found that being involved in a occupation had a greater positive attitude than being unemployed or a housewife [38, 39]. However, in a multiple regression analysis, being students were associated with the more positive attitudes in the present study. In addition, according to the study finding, being divorced showed higher positivity towards COVID-19 outbreak than unmarried and it was similar according to the study conducted in Chinese residents [39].

Likewise, nearly half of the total participants (47.4%) had adverse lifestyle scores, while the rest showed fairly good lifestyle practice. This finding is consistent with several studies conducted in India, China and Iran [40, 41]. Additionally, adverse lifestyle scores were influenced by male gender, having no formal education/lower educational status, and rural residence as per as multiple regression analysis. In agreement with this finding, males are more vulnerable compared to females during the COVID-19 pandemic [42, 43]. Accordingly, a study in Iran also reported that higher level of education provides more practice scores [44]. Several studies provide similar evidence about education plays an influential factor for healthy practice [45, 46]. The present study found a significant association with residence factor which was seen in other studies [47, 48]. The present study found almost 59.6% participants had sufficient storage of food and 52% had enough medication in their home during lockdown which were asked because of the scarcity of supply. It shows consistency with a study conducted in India that demonstrated almost one-third of participants were motivated to buy and stock essential commodities in case of scarcity [49]. Further, studies in Germany and Kashmir valley in India stored sufficient food for more than a week during the pandemic [50, 51]. In addition, a sizeable minority (42.5%) reported a better relationship with their close one during this pandemic, which indicates a significant risk factor for developing a chronic anxiety disorder, and depression among the rest of them [52, 53]. Indeed, quality knowledge along with positive attitude towards the COVID-19 pandemic brings good hygiene practice among people [51, 52]. Overall, appropriate knowledge during a crisis plays a key role for changing individual behavior [54].

The findings of the study predict overall increasing positive attitudes and lifestyle practice towards COVID-19 during lockdown with lifestyle practice needing improvement. This study recommends that the marginalized group of people (i.e., those who don’t have sufficient food or medicine at home), should be provided with necessary and consistent supports since the pandemic doesn’t seem to be abated so quickly. In addition, there is also a need of promoting more quality health education program such as mass education programs both in rural and urban areas.

### Limitations

The present study had several limitations. The same proportion of data could not be collected from all eight divisions of Bangladesh due to the unavailability of spontaneous participants and data collecting volunteers as the country was under complete lockdown during the study period. Also, online data collection was interrupted several times because of limited internet connectivity in some rural areas. Most of the data, including socio-demographic, medical conditions, and lockdown activities were self-reported, so there was no scope to check their authenticity. In the case of some unfortunate and vulnerable people from remote areas, they might be shown to have a negative attitude in most responses due to inaccessibility or unaffordability of available health facilities or other services. However, special attention or support should be given to them for any further research. Furthermore, the present study employed limited factors due to the unavailable information at the beginning of the COVID-19 outbreak that should be warranted in a future study with more comprehensive factors.

## Conclusions

This study conveys the message about how the COVID-19 lockdown has disproportionately impacted the attitudes, activities, and conditions of Bangladesh citizens across various socio-demographic conditions. The analytical outcome of this study should help to understand the response to the COVID-19 lockdown involving respondents from different niches in Bangladesh. The findings from this study should contribute to the optimization and regulation of restrictive measures depending on its necessity and effectiveness on different groups of people in the country. Overall, the study recommends a mass involvement of policymakers, health service providers, stakeholders, and general people to spread knowledge, provide necessary supports and reduce the overall risk of the pandemic like COVID-19 in Bangladesh and also in other low- and middle-income countries.

## Data Availability

All the data generated during the study are presented within the manuscript.
